# Progressive cutaneous Cryptococcosis complicated with meningitis in a myasthenia gravis patient on long-term immunosuppressive therapy – a case report

**DOI:** 10.1186/s12879-017-2415-8

**Published:** 2017-04-26

**Authors:** Nguyen Thi Cam Huong, Ahmed M. A. Altibi, Nguyen My Hoa, Le Anh Tuan, Samar Salman, Sara Morsy, Nguyen Thi Bich Lien, Nguyen Thanh Truong, Nguyen Thi Hoang Mai, Pham Thi Le Hoa, Nguyen Ba Thang, Van The Trung

**Affiliations:** 1 Department of Infectious Diseases, University of Medicine and Pharmacy at Ho Chi Minh City , Ho Chi Minh city, Vietnam; 2 Faculty of Medicine, University of Jordan, Amman, Jordan; 3 Dermatology and Venereology Department, Tanta University Hospital, Tanta, Egypt; 4 Biochemistry Department, Faculty of Medicine, Tanta University, Tanta, Egypt; 5 Ho Chi Minh City Hospital of Dermatology-Venereology, Ho Chi Minh City, Vietnam; 6 The Hospital for Tropical Diseases, Ho Chi Minh City, Vietnam; 7 Department of Dermatology, University of Medicine and Pharmacy at Ho Chi Minh City, Ho Chi Minh City, Vietnam; 8 Oxford University Clinical Research Unit, Ho Chi Minh City, Vietnam; 9 Department of Neurology, University of Medicine and Pharmacy at Ho Chi Minh City, Ho Chi Minh City, Vietnam

**Keywords:** Cryptococcus, Cutaneous Cryptococcosis, Cryptococcus meningitis, Myasthenia gravis

## Abstract

**Background:**

Cryptococcosis is an opportunistic infection caused by the encapsulated yeast *Cryptococcus neoformans* and most remarkably manifests in HIV-infected individuals, especially in the settings of very low CD4 count. Development of cryptococcosis in HIV-uninfected individuals is exceedingly rare and usually signifies a marked immunodeficiency. Cryptococcosis in association with myasthenia gravis or thymoma has been previously documented in only very few cases in the literature.

**Case presentation:**

We reported a complicated case of severe cutaneous cryptococcosis in a 39-year-old Vietnamese male patient with myasthenia gravis on long-term immunosuppressive therapy. The patient presented with a five month history of recurrent and progressive skin lesions that later on progressed into cryptococcal meningitis.

**Conclusion:**

Through this case, we aimed to emphasize the importance of including cutaneous cryptococcosis in the differential diagnosis of cutaneous lesions in patients on chronic immunosuppressive therapy. The cutaneous manifestations of cryptococcosis can be the first clue for a disseminated disease, which makes early recognition crucial and life-saving.

## Background

Cryptococcosis is an infection caused by two species of the encapsulated yeast *Cryptococcus*: *Cryptococcus neoformans* (*C. neoformans*) and *Cryptococcus gatti (C. gatti). C. neoformans* is a rare opportunistic infection that is typically reported causing disease in immunocompromised patients, while *C. gatti* is an endemic infection in the tropical parts of the continent of Africa and Australia and is capable of causing disease in non-immunocompromised individuals.

Cryptococcosis, caused by *C. neoformans*, was known for a long time as an opportunistic infection that occurs exclusively in patients with HIV. Nowadays, many case-studies proved that this pathogen can cause disease in immunocompetent patients as well [1–3] and can also be fatal in patients without known immunosuppression history [3].

The pathogen can cause a wide range of diseases, ranging from isolated cutaneous cryptococcosis to fatal meningoencephalitis and disseminated disease affecting lungs, lymph nodes, skin, and brain [2]. It is transmitted to humans primarily by inhalation of the fungus present in the soil contaminated with avian excreta or decaying wood in the hollows of trees [4]. Other rare methods of transmission have been reported, including organ transplantation and from contact with birds [5, 6].

Although it is well-known for causing meningoencephalitis, pulmonary and cutaneous cryptococcosis were reported in many cases. Cutaneous cryptococcosis is present in 5 % of patients with meningoencephalitis with increasing prevalence in patients with organ transplantation and disseminated disease [7]. However, this is not always the case, cutaneous cryptococcosis has been recently reported in patients with no signs of systemic infection and can present solely as localized cutaneous lesions with positive cultures for *Cryptococcus* species [7]. This predominantly cutaneous presentation is reported in both immunocompetent and immunocompromised patients [1].

Cryptococcosis is also reported in immunocompromised HIV-uninfected patients. This includes patients with organ transplantation, prolonged corticosteroids or immunosuppressive therapy. Cryptococcosis has also been rarely reported in myasthenia gravis (MG) patients.

MG is an autoimmune disease with auto-antibodies that attack specific receptors located on the surface of muscle cells preventing acetylcholine from binding to them, and thus, preventing the muscle from responding to the nerve signal. That is why although treatment with anticholinesterase agents produce some benefit, the prognosis remains poor. More recently, with the use of immunosuppressive therapy or surgical removal of thymus gland the general outlook for MG has improved dramatically [8]. Generalized immune suppression from the MG treatment compromises the immune system as a whole with increased susceptibility to infections, including opportunistic infections, and neoplasia [9–11]. Here, we reported a complicated case of a predominantly cutaneous cryptococcosis that progressed later to meningitis in a myasthenia gravis patient on chronic immunosuppressive therapy.

## Case presentation

A 39-year-old Vietnamese male presented with a five month history (from June to November 2016) of recurrent and progressive skin lesion. Initially, the patient had painless papules distributed at multiple locations, including the chest, arms, hands, thighs, and feet. The papules then progressed into nodules, which later on expanded, ulcerated and became painful.

At the age of 27, the patient was diagnosed with myasthenia gravis (MG), was treated with prednisolone and pyridostigmine since August 2004 and had surgical removal of thymus gland on November 2004. The regimen for his MG from 2010 to 2016 was methyl prednisolone and periodically combined with IMDH inhibitors (mycophenolate or azathioprine) based on his weakness. The starting dose on September 2010 were azathioprine 100 mg/day and methyl prednisolone 48 mg/day. These doses were adjusted afterwards under the consultance of his doctor for 2 years and he did self adjusted later by his objective feeling of weakness. At the time of admission, he was under azathioprine 50 mg/day and methyl prednisolone 16 mg/day.

The patient was also diagnosed with type II diabetes one month after his first eruption (July 2016). Since then, his blood glucose level has been monitored and controlled by dietary modification, his periodically HbA1C were under 6%. From June to September 2016, the patient had completed twelve weeks of directly-acting antivirals (DAAs) for his chronic hepatitis C and he had gained virologic response (HCV RNA negative after 3 months of stop treatment).

Additionally, he had been diagnosed with TB pneumonia at the Pham Ngoc Thach Hospital which is one of the top ranked TB centers in Vietnam for his cough, hemoptysis, shortness of breath, prolonged fever with body temperature from 38 °C - 39 °C, abnormal chest X ray and the Acid-fast bacilli positivity of the sputum. He was started on anti-tuberculosis regimen (2RHZE/4RHE) since September 2016. The regimen consisted of rifampin, isoniazid, pyrazinamide and ethambutol for two months, then withdrawn pyrazinamide for the next four months. His symptoms and chest X ray improved after two weeks of RHZE treatment. He was still on the last week of the first 2RHZE at the time of admission.

**Fig. 1:**
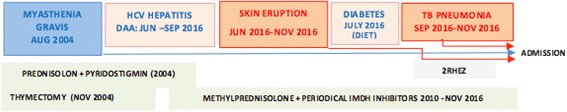
Chronological order of the patient’s medical history

The chronological order of the patient’s medical history is shown in Fig. 1.

It is worth mentioning that the patient had been breeding birds and had close contact with them for the past three years. He had no history of skin trauma within 1 month before the outbreak of painless papules.

On admission, the patient’s main complaints were mild fever, painful and persistent skin lesions with painful and swollen left knee that made him unable to stand or walk. For the past few days, he also had headache that was dull in nature, extended from anterior to posterior and was worse in the afternoon. He also had 5–6 bowel movements/day with loose stool. He did not have symptoms of cough and short breaths during this illness.

He looked thin, pale, anemic, and was only able to sit with support. His temperature was 38.5 °C. He had no neck stiffness, no hepatosplenomegaly or lymphadenopathy. His left leg had intense inflammation (red, swollen, hot, and tender) that resembled cellulitis.

**Fig. 2:**
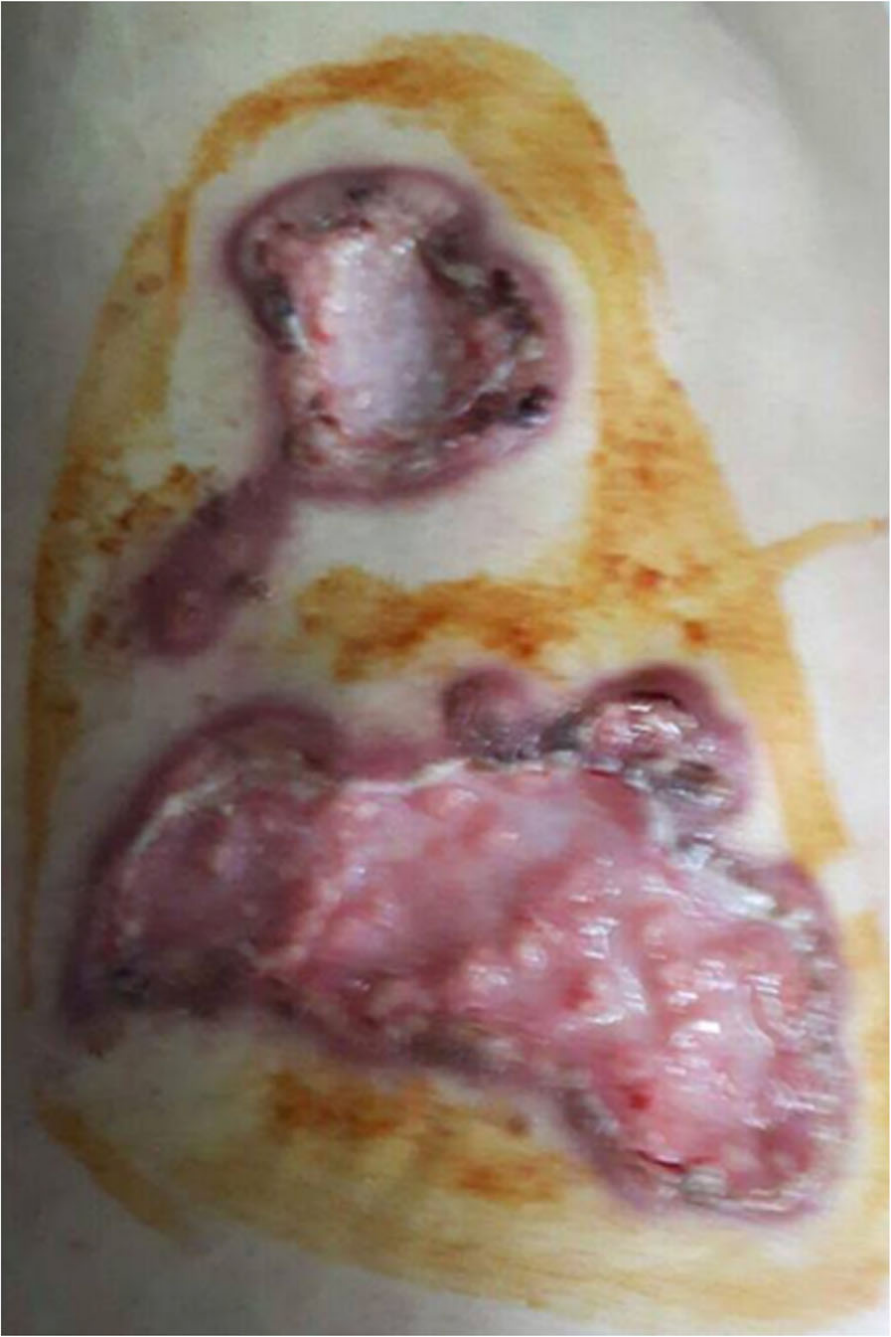
Large, deep, ulcers on the left chest at 1^st^ day of admission. *Cryptococcus neoformans* was detected upon culturing fluids expressed from these ulcers

**Fig. 3:**
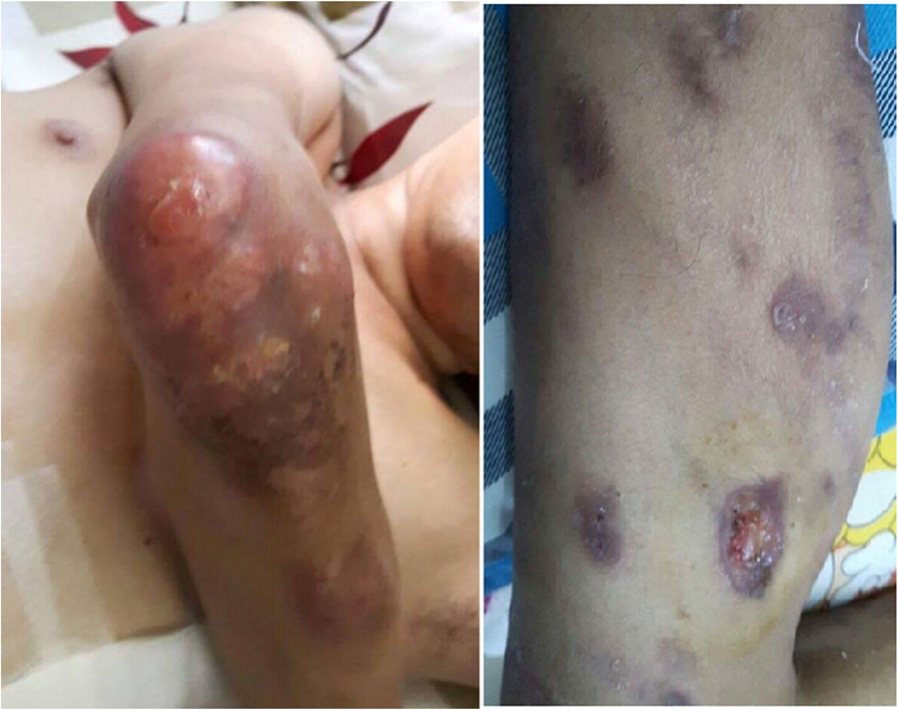
The cutaneous lesions at 1^st^ day of admission: right arm (left) and left leg (right)

**Fig. 4:**
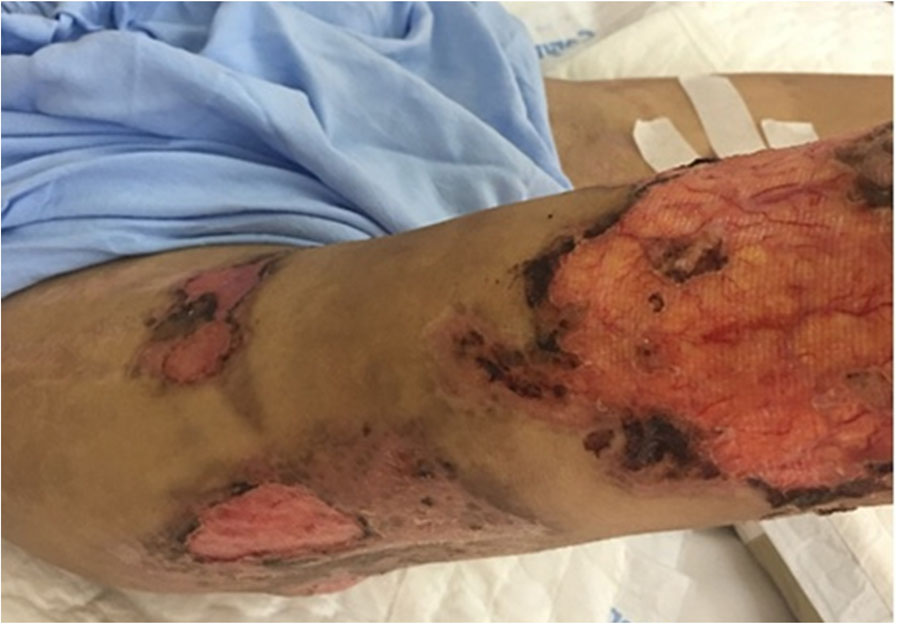
Ulcers on the right thigh at day 11 after admission. The ulcers were deep and with pus and bled when it was cleaned by nurse

He also had multiple ulcers on his body. The 10 × 15 cm ulcer on his left chest (Fig. 2) was deep with visible capillaries at the base, purulent and bleeding. A group of skin lesions 5 × 10 cm was seen on his right arm **(**Fig. 3). There were multiples papules, which some of them progressed into nodules and ulcers (Fig. 3). The largest and deepest ulcer (15 × 20 cm) was on his right thigh (Fig. 4). It was necrotic, purulent, bleeding, and had visible capillaries and small bullous with cloudy fluid in the margins. The presentation of the ulcerative skin lesions simulated Pyoderma gangrenosum.

On complete blood count, the patient was found to have low hemoglobin (7.6 g/dL) and low red blood cells (RBC) count (2.64 M/mL). The white blood cells (WBC) and platelets counts were within the normal ranges. The first blood culture was negative. HIV testing was negative while his CD4 count was remarkably low (236 cells/mL). The MRI of the head did not reveal any lesions in the brain and the chest X-Ray was normal.

The fluids from his bullous skin lesions were collected for microbiology stain and culture. These results came back negative for bacteria. Despite the negative culture, the patient was treated empirically with a combination of meropenem and vancomycin for fourteen days. During the time under antibiotics, the patient was still fever. The headache and skin lesions and were not improved.

**Fig. 5:**
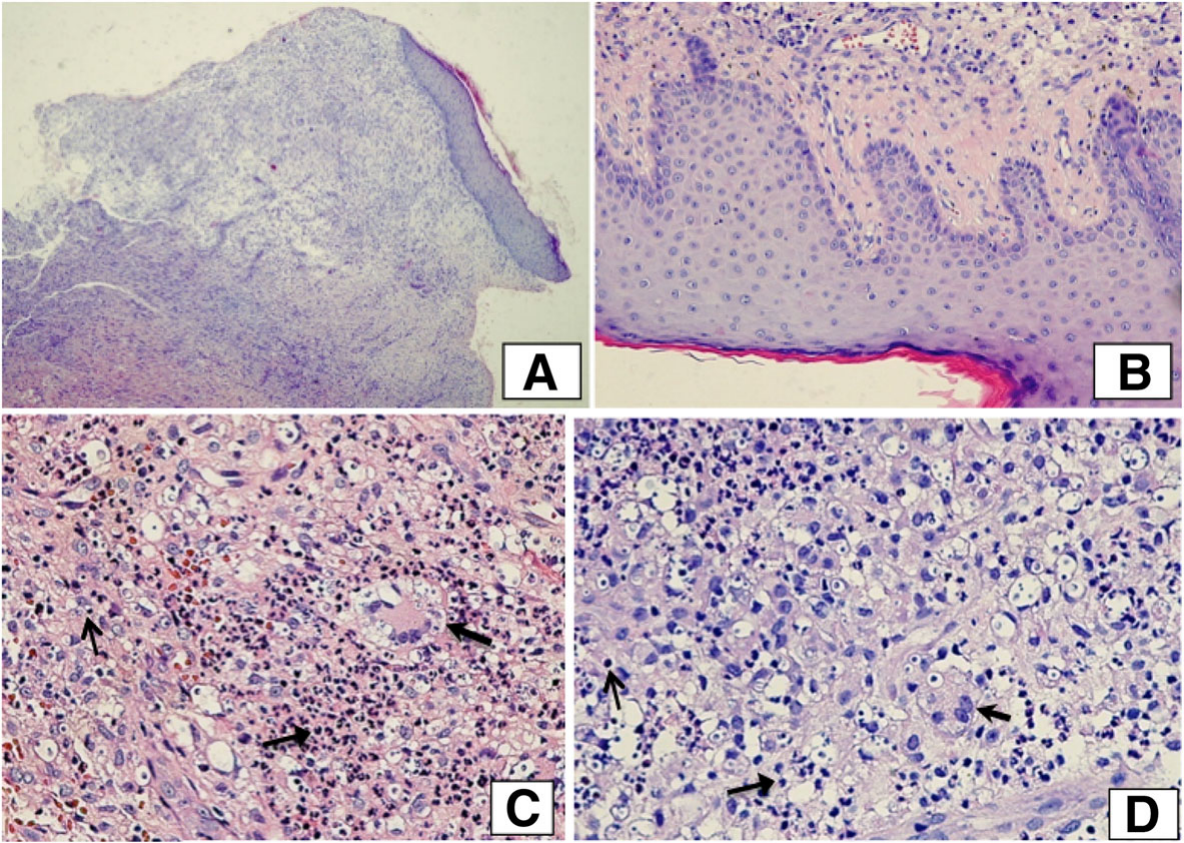
**a** and **b.** Light microscopic view (HE ×200 and HE ×400) of ulcer tissue from chest and leg shows loss of the epidermis and some portions of the dermis and subcutaneous fat. Lymphocyte and macrophages infiltrates with neutrophiles. **c** and **d:** The dermal infiltrates consisted of lymphocytes (**↑**) and a few monocytes-macrophages ; and numerous neutrophils  created sporadic focal abscesses

**Fig. 6:**
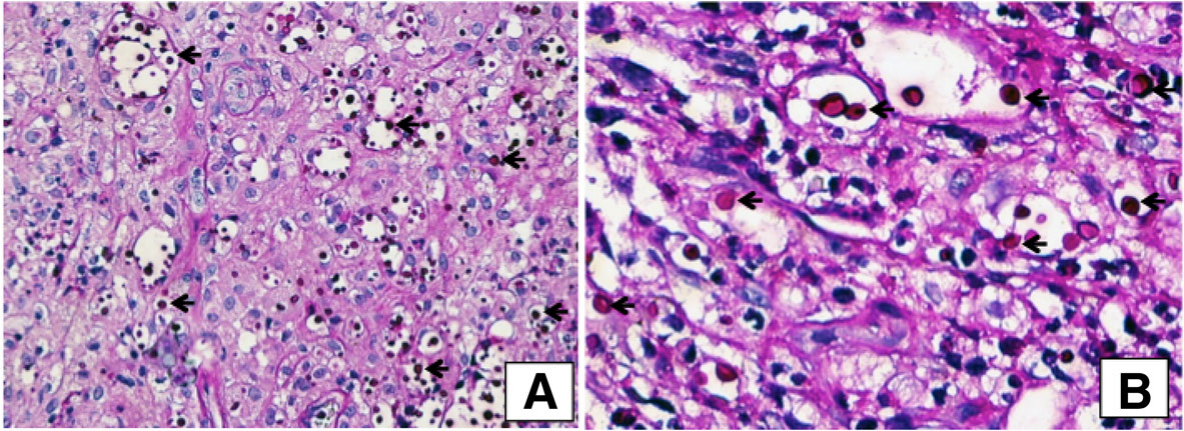
**a** and **b.** PAS-stained (PAS ×400 & PAS ×1000) showing numerous Cryptococcal spores inside macrophages (←) that stain positive with PAS. *Ziehl-Neelson stain was negative for acid-fast bacilli*

The skin biopsy of the chest and leg ulcers was performed subsequently. The histopathological results detected the spores of cryptococcosis inside the macrophages (Figs. 5 and 6).

The patient underwent a lumbar puncture at admission. The cerebral spinal fluid opening pressure was 9 cm H_2_0 and all the opening pressures afterwards were normal. The analysis of the cerebral spinal fluid (CSF) detected elevated protein levels (1.61 g/L), mildly decreased glucose level (2.14 mmol/L, serum glucose: 4.84 mmol/L), lactate of 3.76 mmol/L and a white cell count of 112 cells/μL with neutrophilic predominance (78%). Lateral flow assay (LFA) test of the CSF was positive for cryptococcal antigen. The CSF cultures, however, were negative for both bacteria and fungi. Hence, the second culture of the smear of the skin ulcers was performed and was positive for *C. neoformans* and negative for bacteria.

Subsequently, the patient was started on amphotericin B (1 mg/kg/day) and fluconazole (800 mg/day) firstly in parallel with methyl prednisolone, azathioprine, pyridostigmine and anti-tuberculosis regimen. As planned, amphotericin B and fluconazole would be continued for 28 day. Fluconazole, for consolidation, would be continued for 11 months later.

The fever subsided after 4 days of treatment with amphotericin B and fluconazole but the patient still complained a severe headache. The neurologist considered the immune suppression effect of azathioprine, the stable status of MG and the low CD4 cell count and decided stopping azathioprine and lowered the methyl prednisolone dose to 4 mg/day for better control of Cryptococcal infection. The patient’s hypokalemia, attributed to amphotericin B, was also corrected via oral potassium replacement.

A dramatic clinical and microbiologic improvement was observed after 11 days of treatment: the anemic status improved with hemoglobin level increasing to 9.0 g/dL without RBC transfusion, the headache subsided, the meningeal signs disappeared and the patient became afebrile. The ulcers became smaller, with no exudates, granulated and improved quickly. The culture of skin ulcers on day 10 post-treatment was negative for both bacteria and fungi.

On day 14 of treatment, the WBC count of the CSF dropped to 19 cells/mL and the LFA test became negative; the glucose level was 3.53 mmol/L (serum glucose: 10.87 mmol/L) and the protein remained high (1.66 g/L).

On day 28 of treatment, the WBC count of the CSF was 12 cells/mL, the protein was 1.34 g/L, the glucose was 2.82 mmol/L (serum glucose: 6.02 mmol/L). The lactate level dropped to 2.48 mmol/L but the LFA test was positive again although the fungus culture was still negative in the CSF. The skin ulcer cultures were negative also for both bacteria and fungi.

After 28 days of treatment, amphotericin B was stopped and fluconazole (1000 mg) was consolidated until 12 months of treatment as recommended. We used a high dose of fluconazole because the patient was using rifamycin (anti-tuberculosis) which is known to reduce the level of fluconazole.

**Fig. 7:**
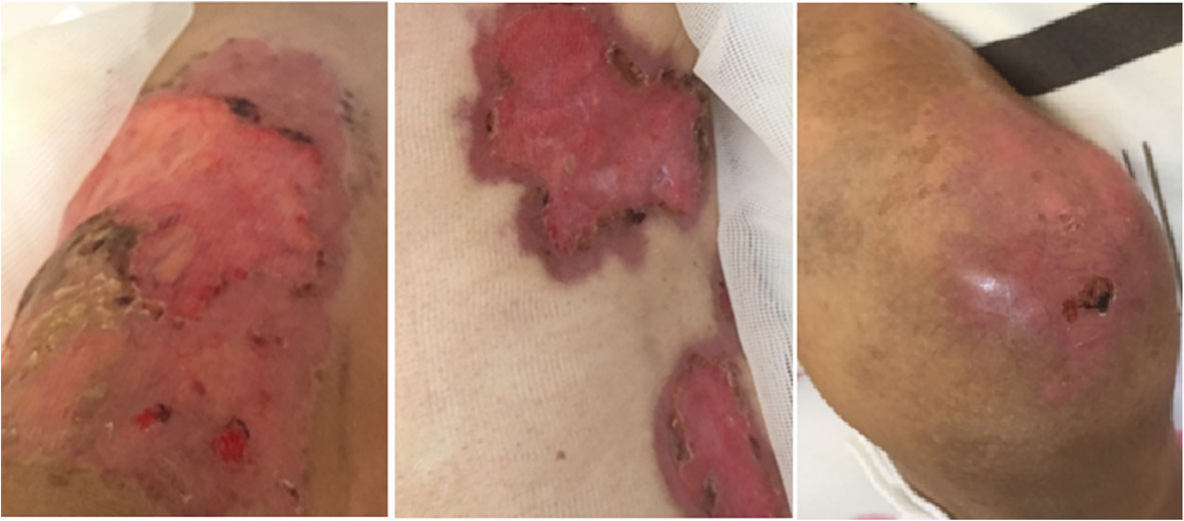
Cutaneous lesions at the day of discharge: Left chest (middle), right thigh (left) and right knee (right): these lesions were reduced in size compared to initial lesions

The patient was finally discharged on day 32. He had also been advised to avoid contact with birds. The cutaneous lesions on the day of discharge are shown on (Fig. 7). The patient will be re-examined every two months during the time of fluconazole maintenance.

## Discussion

Cryptococcosis in HIV-uninfected patients almost always develops in the setting of immunocompromised status, such as patient with organ transplant, with malignancies or on chronic immunosuppressive treatment (e.g., corticosteroids) [12]. The development of cutaneous cryptococcosis is exceedingly rare in HIV-uninfected population.

Here, our patient has multiple risk factors. He has been on prolonged immunosuppressive therapy and had a low CD4^+^ cell count (236 cells/mL). Additionally, he had also been diagnosed with diabetes mellitus which is considered a clue for immune dysfunction [13]. Moreover, the patient also had tuberculosis, which most likely resulted from reactivation of a latent infection, another warning for weakening of the immune system. All of these coexisting factors in this patient revealed a high susceptibility to opportunistic agents such as *Cryptococcus neoformans*.

Our patient had a prolonged course of cutaneous lesions (more than 5 months) before the final diagnoses was figured out. This can be justified by the great variation in cutaneous cryptococcus presentations and the rarity of this kind of infection in HIV-uninfected patients.

Additionally, in our case the initial bacterial culture of the fluid from the skin lesions was negative. Nevertheless, by the time of histopathological detecting of spores from the ulcer, the repeated culture of the smear from skin lesion for both fungal and bacteria was also positive for *Cryptococcus*. This adds to the complexity of making the diagnosis and provides an additional evidence that histopathology has a core role in making a rapid diagnosis of the disease – as many times the culture fails to flourish the pathogen [14]. The culture of the bullous fluid or smears from the ulcers was more sensitive for bacterial pathogens, but less helpful for fungal pathogens, especially when suspected fungal pathogens were not specifically notified by the clinician.

From this case, we recommended physicians to perform a culture for yeast in all skin lesions in immunosuppressed patients or to perform a Lateral Flow Assay (LFA). The cryptococcal antigen LFA is a rapid, very sensitive, easy-to-perform assay with a demonstrated usefulness as a point-of-care assay for diagnosing cryptococcosis in resource-limited countries. The availability of this assay in remote locations could have a meaningful impact on early diagnosis of cryptococcosis [15].

Despite being reported many times in the literature, arguments continue on the existence of primary cutaneous cryptococcosis versus cutaneous cryptococcosis being only secondary to hematogenous dissemination [16].

The patient had no history of skin trauma but he had bred birds many months in the past. He could had skin cuts once or several times without his notification. We could not precisely conclude whether he was acquired the cryptococcus through skin lesion or through respiratory aspiration of Cryptococcal spores. However, in case of aspiration the chronological sequence should be: firstly presentation of lung infection with respiratory symptoms and signs, bacteremia later, and lastly meningitis or skin appearances. His true progression was reversed with the skin ulcer were found several months in advance, the meningeal affect (headache, fever) progressed much later after the presentation of skin lesions, and the lung was not been affected at the time of menigitis. Therefore, the patient should be a case of primary cutaneous Cryptococosis, circulatory disseminated and secondary menigitis.

It is worth mentioning that, similar to our case, systemic or disseminated infections can firstly present solely with skin lesions and later progress to involve other organs such as the meninges [17]. Hence, a high index of suspicion is required to reach to an early diagnosis.

The treatment recommendation for HIV-uninfected patients who are diagnosed with cryptococcal meningoencephalitis is to give induction therapy with amphotericin B for 2–4 weeks followed by consolidation with fluconazole (400–800 mg/day) for 12 months [12]. In our case, however, after induction with Amphotericin B, we adjusted the dose of fluconazole into 900–1000 mg/day as the patient was receiving rifampicin which is known to reduce the blood level of fluconazole [18].

In case of cryptococcosis persisted during treatment or recurred after sufficient therapy, then we should consider antifungal resistance, relapse or immune reconstitution inflammatory syndrome. Resistance, that comes if we never achieve CSF sterility, could occur with low dose of fluconazole, especially if it is used as a monotherapy. We could deal with relapse by re-induction therapy with amphotericin at high doses (1 mg/kg/day) for at least seven days and fluconazole dose could be increased up to 1200 mg/day [19]. On the other hand, sudden onset deterioration with worsening meningitis symptoms along with CSF sterility or stable titers of cryptococcal antigen support the diagnosis of immune reconstitution inflammatory syndrome [20].

This is not the first case in literature to report cryptococcosis in patients with MG [8–11, 21–23]. And although not surprising in a MG patient on chronic immunosuppression, this is only the eighth case reporting on such association. Infections in previously reported cases were in the forms of meningitis in three cases [8, 9, 11], prostatic abscess in one case [21], prosthetic joint infection in one case [22], cryptococcal cellulitis [10] in one case and disseminated cryptococcal infection in one case [23].

However, despite the fact that patients with diseases of the lymphatic systems are prone to fungal infections [11], this simultaneous occurrence of cryptococcosis and MG could be only a pure coincidence and directly attributed to the steroids-induced immunosuppression status.

## Conclusion

This case is an opportunity to emphasize on the importance of including cutaneous cryptococcosis in the differential diagnosis of cutaneous lesions in patients on chronic immunosuppressive therapy, especially if the skin lesions are resistant to empiric antibiotic treatment. The cutaneous manifestations of cryptococcosis can be the first clue for a disseminated disease, which makes early recognition crucial and life-saving.

## Abbreviations

*C. gatti*: *Cryptococcus gatti*
*C. neoformans*: *Cryptococcus neoformans.*
CSF: Cerebral spinal fluid.
DAAs: Directly-acting antivirals.
LFA: Lateral flow assay.
MG: Myasthenia gravis.
RBC: Red blood cells.
WBC: White blood cells.
